# Radiation dose estimation with time-since-exposure uncertainty using the $\gamma$-H2AX biomarker

**DOI:** 10.1038/s41598-022-24331-1

**Published:** 2022-11-18

**Authors:** Dorota Młynarczyk, Pedro Puig, Carmen Armero, Virgilio Gómez-Rubio, Joan F. Barquinero, Mònica Pujol-Canadell

**Affiliations:** 1 Departament de Matemàtiques, Universitat Autònoma de Barcelona, Bellaterra, Spain; 2 Centre de Recerca Matemàtica, Bellaterra, Spain; 3 Departament d’Estadística i Investigació Operativa, Universitat de València, València, Spain; 4 Department of Mathematics, School of Industrial Engineering, Universidad de Castilla-La Mancha, Albacete, Spain; 5 Departament de Biologia Animal, Biologia Vegetal i Ecologia, Universitat Autònoma de Barcelona, Bellaterra, Spain

**Keywords:** Biomarkers, Statistical methods, Statistics

## Abstract

To predict the health effects of accidental or therapeutic radiation exposure, one must estimate the radiation dose that person received. A well-known ionising radiation biomarker, phosphorylated $\gamma$-H2AX protein, is used to evaluate cell damage and is thus suitable for the dose estimation process. In this paper, we present new Bayesian methods that, in contrast to approaches where estimation is carried out at predetermined post-irradiation times, allow for uncertainty regarding the time since radiation exposure and, as a result, produce more precise results. We also use the Laplace approximation method, which drastically cuts down on the time needed to get results. Real data are used to illustrate the methods, and analyses indicate that the models might be a practical choice for the $\gamma$-H2AX biomarker dose estimation process.

## Introduction

Ionising radiation is currently used for a variety of purposes, such as industrial radiography, energy production, and health diagnosis and treatment. This results in an increased risk of radiological accidents. When a radiological accident occurs, it is important to assess the dose of radiation absorbed by people affected to help decision-making measures and whether it is necessary to help with medical care. Biodosimetry refers to the use of several biological markers to quantify the exposure to radiation of any person suspected of being exposed. The present study focuses on the $\gamma$-H2AX protein, which is an accepted biomarker of dose exposure to ionising radiation^1^.

**Figure 1:**
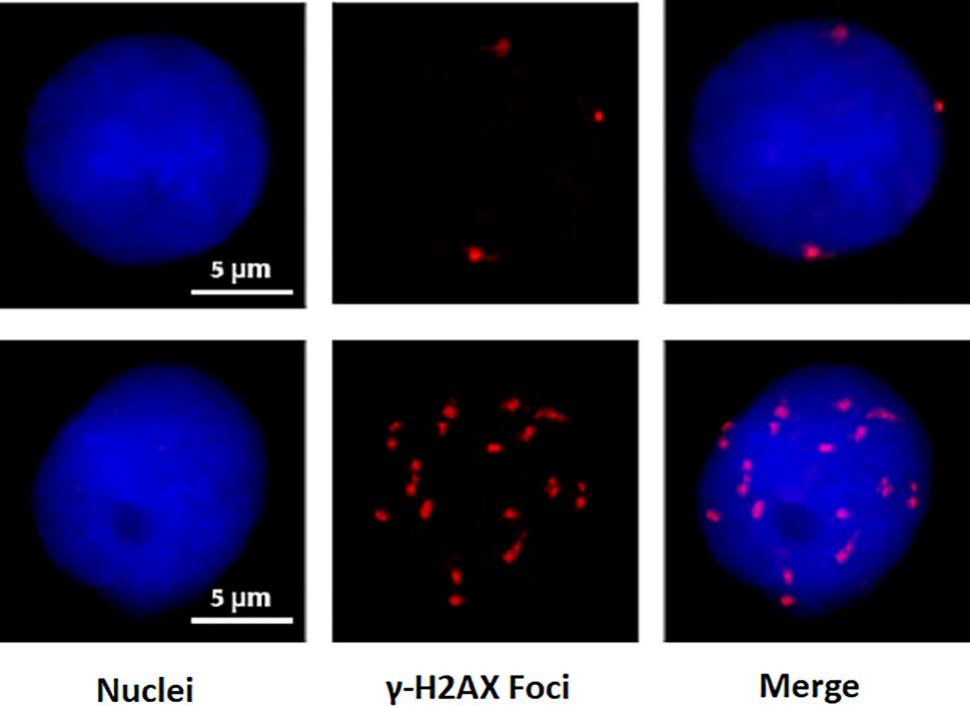
Images of nuclei from peripheral blood mononucleated cells stained with 4$\prime$,6-diamidino-2-phenylindole (DAPI) in blue, containing foci of $\gamma$-H2AX immunostained with Cyanine 3 (Cy3) in red.

When a cell is exposed to radiation, a wide variety of DNA damage can occur, including double-strand breaks (DSBs), which literally means that the two strands of the double helix break in close proximity. The cell has DNA damage response mechanisms, that they are activated in the face of this type of disruption. A critical modification is that in presence of DSBs the histone variant H2AX is phosphorylated ($\gamma$-H2AX) near the break site. This modification is extended to many megabases of the chromatin allowing to be detected microscopically in form of nuclear foci by immunostaining techniques^2^, as seen in Fig. 1. Those visible fluorescent foci can then be scored manually or automatically by a computer program. Because foci yield is proportional to the absorbed radiation dose, the results from several hundred cells can be used for dose estimation analysis^1,3^.

The first part of the dose estimation method involves the calibration process. Samples of human peripheral blood lymphocytes are irradiated under controlled conditions at predetermined doses. Then the number of foci counts in peripheral blood mononucleated cells (PBMCs) is collected and the results are fitted to regression curves or surfaces, giving a calibration model. To determine the absorbed radiation dose by an exposed person, the number of $\gamma$-H2AX foci in the blood sample of that person must be evaluated. Then an inverse regression method^4,5^, using the calibration model and the foci counts in the patient’s sample, allows to estimate the dose received by the patient.

**Figure 2:**
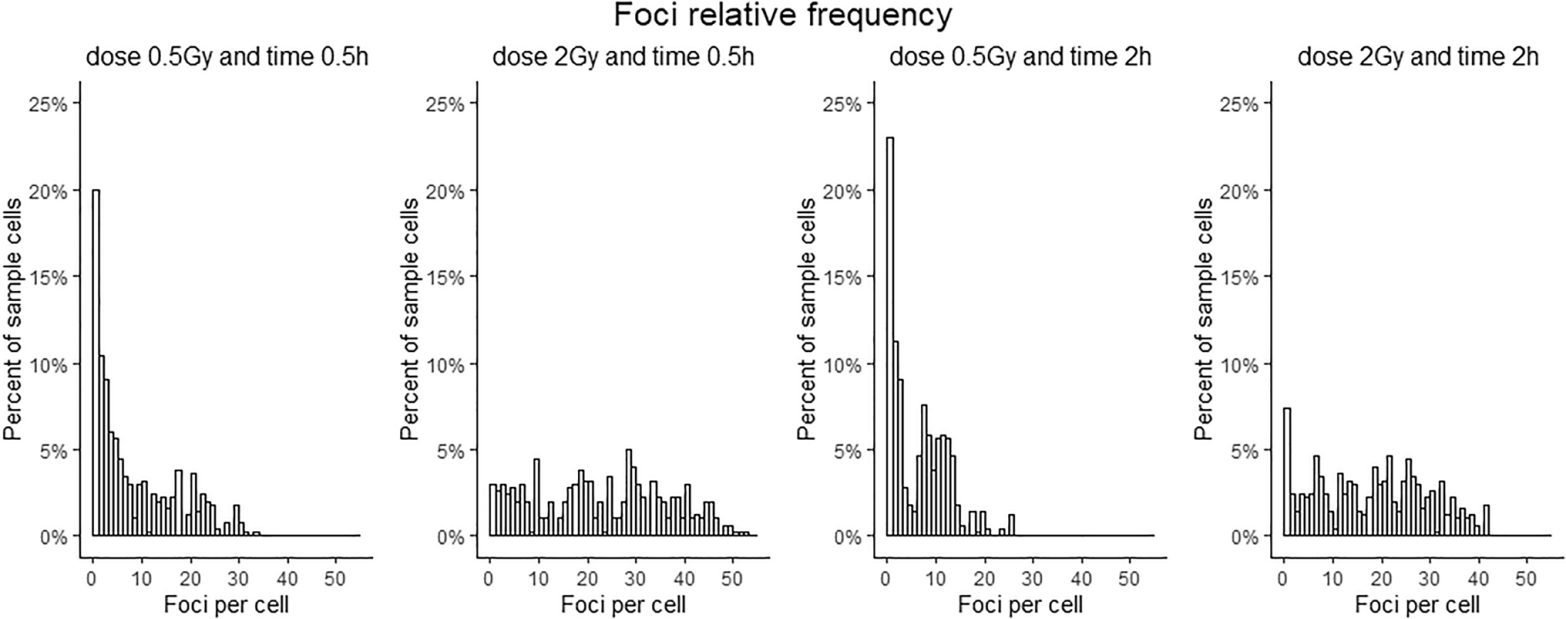
$\gamma$-H2AX foci relative frequency found in 500 cells for four different scenarios (from left to right): at 0.5 Gy and 0.5 h, at 2 Gy and 0.5 h, at 0.5 Gy and 2 h, at 2 Gy and 2 h.

It is known that the number of DSBs depends on the amount of radiation received by cells; the more radiation absorbed, the greater the biological damage. However, the $\gamma$-H2AX foci are influenced not only by dose but also by the time from exposure^6^. As studied before, the $\gamma$-H2AX level reaches the maximum 30 min after exposure and then gradually decreases over hours as the cells repair the damage, returning to normal levels within 24–48 h^7,8^. For this reason, in the case of a radiological accident the utility of $\gamma$-H2AX assay is restricted to rapid usage. The distribution of the number of observed foci varies greatly depending on the time and dose, as shown in the Fig. 2. As the dose is increased, fewer zeros appear because cells tend to have more foci. The number of foci, on the other hand, gets smaller over time.

Due to the great variability over time of the foci data, the calibration curves have commonly been constructed separately for different times after exposure^9,10^. From a practical point of view, this approach is somewhat unrealistic given the continuity of time and the inability to accurately determine the exact time point of irradiation. Therefore, it seems a noteworthy idea to include the time as a variable in a calibration model and to consider a three-dimensional response surface, i.e. simultaneously dose and time dependent, what was recently proposed by López et al.^11^.

Foci $\gamma$-H2AX data are overdispersed^3,9^, which means that the variance in the observed samples is higher than its mean. Due to this fact, the Poisson model, commonly used in the analysis of count data, may not be appropriate. Instead, many distributions were already proposed, in particular, negative binomial, zero-inflated Poisson and zero-inflated negative binomial models provide good results for $\gamma$-H2AX foci^12^. $\gamma$-H2AX is analyzed in PBMCs, that is composed by a mixture of various types of leukocytes, but different subsets of blood cells could have different levels of $\gamma$-H2AX foci. It has been suggested that the subset of CD4+ leukocytes show 1.5 times higher level of phosphorylation than CD19+^13^. Although this statement requires more investigation because more evidence is necessary, it can be a plausible explanation for the variability in the distribution of foci, hence we opted to use a mixed Poisson model in this paper. Mixture models are a useful tool for modelling data that are highly diverse, and it is thought that this behaviour is due to underlying sub-populations^14^. These models assume that it is not known to which subgroup a particular observation belongs, so a mixture of a few Poisson distributions with different proportions is used to describe all the data.

Moreover, many unknowns may arise in the dose estimation process, such as the impossibility of determining the exact time of irradiation or the individual response to radiation. In this scenario, the Bayesian framework may be more appropriate because it can handle many levels of uncertainty^15^. The Bayesian approach also allows current information to be incorporated into the inferential process, as may be the case in biodosimetry, for example when laboratories prepare a calibration curve (or surface) independent of emergency data. Therefore, some information about the distribution of the model parameters may be provided in advance of a possible accident.

This study aims to investigate the use of biomarker $\gamma$-H2AX in the estimation of the radiation dose absorbed by an individual taking into account the uncertainty on their exposure time. In the second section, we introduce statistical strategies for dealing with the issues outlined before. In the third section, we apply these methods to $\gamma$-H2AX real data and we present the results. In the last part, we discuss some limitations of the statistical proposals and we discuss some directions for further research.

## Statistical models

**Figure 3:**
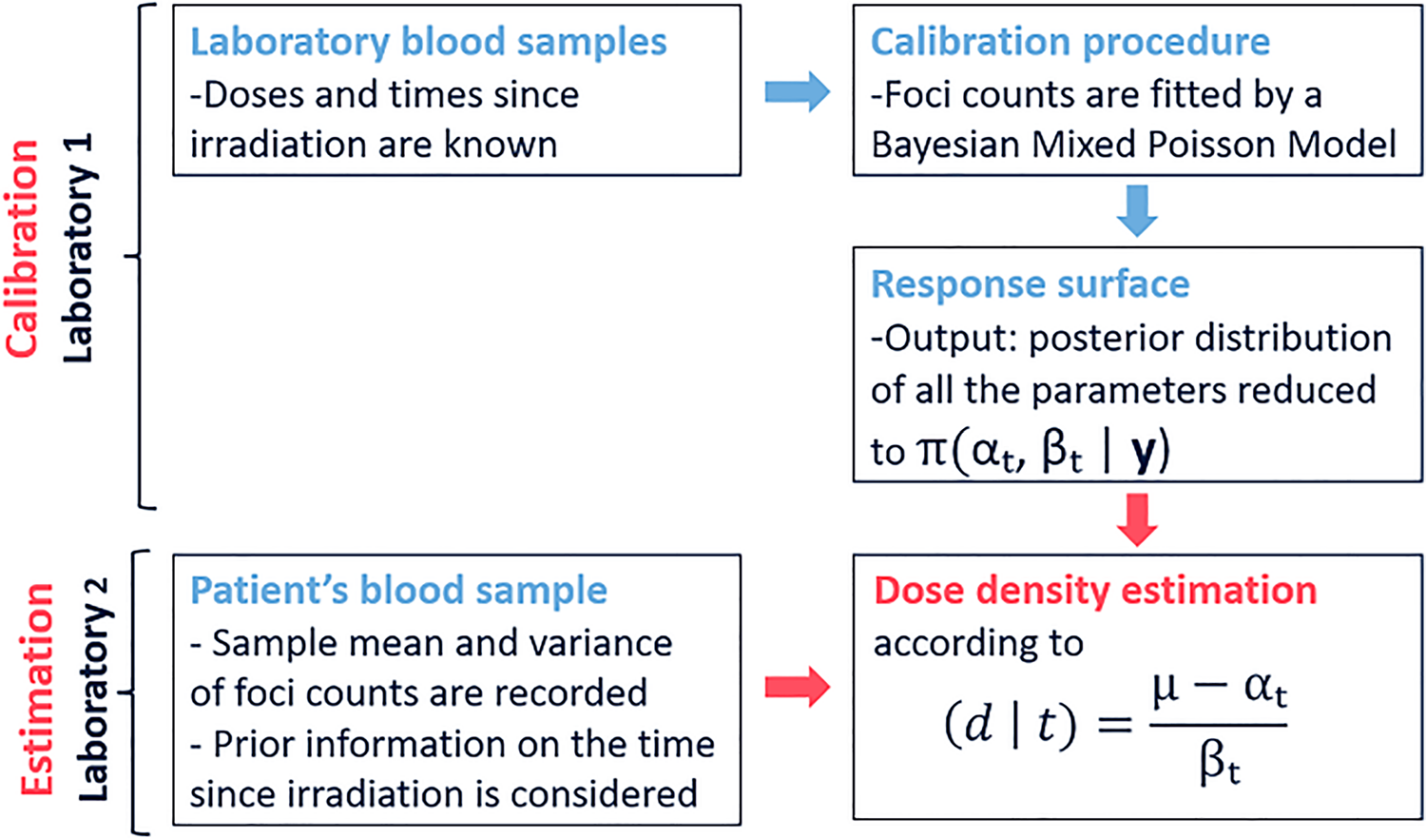
Scheme of the whole dose estimation procedure.

In this section, we describe the proposed statistical methodology for analysing biodosimetric foci-data. The process is divided into two parts: calibration and estimation. The calibration procedure is based on sample laboratory data, implying that the irradiation exposure occurs under monitored conditions. Calibration data were obtained irradiating *N* peripheral mononucleated cells from one donor with radiation doses ranging from 0 to 3 Gy, and $\gamma$-H2AX foci were detected microscopically using a semi-automatic method, at different post-irradiation times from 0.5 to 24 h. The number of $\gamma$-H2AX foci, $y_i$, was recorded, obtaining a set of data ${\textbf {y}}=(y_1, \ldots , y_N)$, assuming conditional independence among observations. The data can be found in the Supplementary material of the paper by López et al.^11^. These data, which serve as a type of reference for how cells behave to different doses and time points, are fitted with our statistical model to create a calibration surface. Then, blood samples from a potentially newly irradiated patient are explored, and the mean and variance of the number of foci counts in *n* cells are recorded. In the estimation part of the process, they are used to estimate the doses supposedly received by those irradiated persons. Calibration is only required once, and the same surface response can be used to analyse many patient samples. In fact, different laboratories can perform calibration and estimation; Fig. 3 describes the process.

### Calibration

As explained in the introduction, we propose a Poisson mixture model with K components to describe the sampling distribution of the number of $\gamma$-H2AX foci per cell. We assume that observations ${\textbf {y}}=(y_1, \ldots , y_N)$ are conditionally independent and have been generated by the following finite mixture

1 $$\begin{aligned} (y_i \mid \varvec{\omega }, \varvec{\lambda }_i) \sim \sum _{k=1}^K \omega _k f(y_i \mid \lambda _{ki}), \end{aligned}$$

where $\varvec{\omega }= (\omega _1, \ldots , \omega _K)$, $\varvec{\lambda }_i=(\lambda _{1i}, \ldots , \lambda _{Ki})$ and $f(y_i \mid \lambda _{ki})= \frac{e^{-\lambda _{ki}} \lambda _{ki}^{y_i}}{y_i !}$ indicates the conditional Poisson probability of observing $y_i$ foci given $\lambda _{ki}$. The parameters $0<\omega _k<1$ represent the weight of each component of the mixture and they sum up to 1, $\sum _{k=1}^K \omega _k=1$.

In general, the parameter $\lambda$ of a Poisson random variable can be modelled in many ways using different link functions. As mentioned earlier, time since radiation exposure has a substantial influence on the presence of the number of $\gamma$-H2AX foci. For this reason, and in line with the model proposed by^11^, $\lambda$ could be defined as a function of two variables, dose and time (represented by *d* and *t* respectively), $\lambda =\lambda (d,t)= c \cdot t^{u} + a \cdot t^{v} \cdot d,$ where (*a*, *c*, *u*, *v*) are parameters. Therefore, $\lambda$ is a three-dimensional surface, although it is important to note that if time is fixed, $\lambda$ is linear with respect to *d*, which is consistent with previous findings^9^.

For the mixture model with *K* components in (1), the parameters $\lambda _{ki}$ are defined separately for each Poisson component $k \in \{1,\ldots , K \}$, $\lambda _{ki}(d,t) = c_k \cdot t_i^{u_k} + a_k \cdot t_i^{v_k} \cdot d_i$ for $i={1, \ldots , N}$. Since the calibration phase uses laboratory data, for a given observed number of foci $y_i$, $t_i$ (time) and $d_i$ (dose) are known. From now on to simplify the notation, $\varvec{\theta }_k=(a_k,c_k,u_k,v_k)$ stands for the set of parameters for component *k* and $\varvec{\theta }=(\theta _1,\ldots ,\theta _K)$ for all parameters in the model. In order to find the calibration surface, these parameters, together with the weights of the mixture $\varvec{\omega }=\{\omega _1,\ldots ,\omega _K\}$, should be estimated, what we propose to do within Bayesian framework.

In the Bayesian schema, all previous information about the quantities of interest are used to elicit a prior density distribution $\pi (\varvec{\omega }, \varvec{\theta })$ for the parameters $(\varvec{\omega }, \varvec{\theta })$. Next, the data ${\textbf {y}}$ are registered and the likelihood $\mathcal L({\textbf {y}} \mid \varvec{\omega }, \varvec{\theta })$ of $(\varvec{\omega }, \varvec{\theta })$ for the data are constructed. In our case, given the conditional independence of the observations $y_i, \ldots , y_N$, the likelihood function remains,

$$\begin{aligned} \mathcal L ({\textbf {y}} \mid \varvec{\omega }, \varvec{\theta })= \prod _{i=1}^{N} \sum _{k=1}^K \omega _k f(y_i \mid c_k \cdot t_i^{u_k} + a_k \cdot t_i^{v_k} \cdot d_i). \end{aligned}$$

Then the Bayes theorem provides the posterior density distribution $\pi (\varvec{\omega }, \varvec{\theta }\mid {\textbf {y}})$:

$$\begin{aligned} \pi (\varvec{\omega }, \varvec{\theta }\mid {\textbf {y}}) \propto&\mathcal L({\textbf {y}} \mid \varvec{\omega }, \varvec{\theta }) \pi (\varvec{\omega }, \varvec{\theta }). \end{aligned}$$

As stated before, Bayesian modelling requires the specification of a prior distribution $\pi (\varvec{\omega }, \varvec{\theta })$. We assume prior independence between $\varvec{\omega }$ and $\varvec{\theta }$ and, consequently $\pi (\varvec{\omega }, \varvec{\theta })= \pi (\varvec{\omega }) \pi (\varvec{\theta })$. We propose to consider non-informative uniform prior distributions for $\varvec{\theta }$, as they will produce a minimal influence on the inference. For $\varvec{\omega }$, we decided to use a non-informative symmetric Dirichlet distribution called Perks’ prior (see Ref.^16^ and Supplementary material).

The analytical derivation of the posterior distribution is intractable due to model’s complexity; however, the Bayesian framework provides numerous computational methods for approximating posterior distributions. In particular, sampling algorithms, which rely on Markov chain Monte Carlo (MCMC) methods, can give a good approximation of the posterior distribution. One such method is the Gibbs sampler, in which samples from $\pi (\varvec{\omega }, \varvec{\theta }\mid {\textbf {y}})$ are constructed from the conditional posterior distribution of each element in $(\varvec{\omega },\varvec{\theta })$ given the rest of them. Posterior estimates of $(\varvec{\omega }, \varvec{\theta })$ are the subsequent posterior means, approximated by the corresponding sample means of the MCMC posterior distribution.

Nonetheless, when dealing with a large number of model parameters, MCMC techniques are often extremely slow. If the absorbed radiation dose must be estimated immediately the technique of providing the results must be as quick as possible. Therefore, we propose to use the Laplace approximation method, which aims to find a Gaussian approximation to the posterior distribution, which results in a faster execution of the analysis. Assume that $\varvec{\phi }^{*}=(\hat{\omega },\hat{\theta }) \in \textbf{R}^p$ is the mode of $\pi (\varvec{\omega }, \varvec{\theta }\mid {\textbf {y}})$, i.e.

$$\begin{aligned} \phi ^{*} = \underset{(\omega ,\theta )}{\arg \max }\ \, \mathcal L({\textbf {y}} \mid \varvec{\omega }, \varvec{\theta }) \pi (\varvec{\omega }, \varvec{\theta }). \end{aligned}$$

The Laplace approximation of the posterior $\pi (\varvec{\omega }, \varvec{\theta }\mid {\textbf {y}})$ provides a *p*-dimensional multivariate normal distribution (details can be found in Supplementary material):

2 $$\begin{aligned} \pi (\varvec{\omega }, \varvec{\theta }\mid {\textbf {y}}) \sim \mathcal {N}_p (\varvec{\phi }^{*}, \hat{\Sigma }_{\hat{\omega },\hat{\theta }}), \end{aligned}$$

where $\hat{\Sigma }_{\hat{\omega },\hat{\theta }}$ is the inverse of the Hessian matrix of the model evaluated at $(\hat{\omega },\hat{\theta })$ (the estimated variance-covariance matrix). The estimated parameters $(\hat{\omega },\hat{\theta })$ will be called *calibration coefficients*.

For a fixed time *t* the expected number of foci provided by the calibration surface remains,

3 $$\begin{aligned} \mu (d \mid t)=\sum _{k=1}^K \omega _k \lambda _{k}=\sum _{k=1}^K \omega _k (c_k \cdot t^{u_k} + a_k \cdot t^{v_k} \cdot d)=\alpha _t +\beta _t d, \end{aligned}$$

where,

$$\begin{aligned} \alpha _t= \sum _{k=1}^K \omega _k \cdot c_k \cdot t^{u_k}=g_1(\varvec{\omega }, \varvec{\theta }),  \beta _t= \sum _{k=1}^K \omega _k \cdot a_k \cdot t^{v_k}=g_2(\varvec{\omega }, \varvec{\theta }). \end{aligned}$$

In (3), additional factors, such as age or gender, might be added as covariates if they were thought to be significant for dose estimation.

The derived posterior distribution of the two time-dependent parameters $(\alpha _t, \beta _t)$ is the output of the calibration process. Using the multivariate delta method, it is straightforward to see that $(\alpha _t, \beta _t)$ follows an approximated bivariate normal distribution,

4 $$\begin{aligned} \pi (\alpha _t, \beta _t \mid {\textbf {y}}) \dot{\sim } \mathcal {N}_2 ({\textbf {g}}(\hat{\omega }, \hat{\theta }), \nabla {\textbf {g}} \cdot \hat{\Sigma }_{\hat{\omega },\hat{\theta }} \cdot \nabla {\textbf {g}}^T), \end{aligned}$$

where $\nabla {\textbf {g}}$ is the gradient of ${\textbf {g}}=(g_1,g_2)$ evaluated at the estimated modal point $(\hat{\omega },\hat{\theta })$.

### Estimation

In this part, we assume that there is available a set of new observations ${\textbf {x}}=(x_{1},\ldots , x_{n})$, coming from a new patient, called test data. In the event of a radiation emergency, these will be the counts of foci detected in the exposed person’s blood cells, for which the absorbed dose must be estimated. After a fast exploration the laboratory records the mean number of foci in the test data $\bar{x}$ and its standard deviation *s*. We can assume that the expected number $\mu$ of foci of the patient, independently of time t, follows a normal distribution $\mu \sim \mathcal {N}(\bar{x}, \frac{s^2}{n})$, where *n* is the number of observations in the test data. This is a kind of nonparametric Bayesian estimate of $\mu$ justified in the Supplementary material.

We use the dose relation described in (3) to relate the patient’s observed foci distribution with time *t* and dose *d*. Then, the dose can be directly determined as

5 $$\begin{aligned} (d \mid t)=\frac{\mu -\alpha _t}{\beta _t}, \end{aligned}$$

where $(d \mid t)$ means that dose is defined for a given value of *t*. Expression (5) establishes that, when the bivariate normal approximation (4) is used for approximating the posterior distribution for $(\alpha _t, \beta _t)$, the posterior distribution of *d* conditioned to *t*, $\pi (d \mid t,{\textbf {y}}, {\textbf {x}})$ can be approximated by the ratio of two dependent normal variables (see Ref.^17^ and Supplementary material). Given a prior information on the post-irradiation time, summarized with the prior distribution $\pi (t)$, the joint posterior density of the time and dose of the new irradiated person can be expresses as $\pi (d,t \mid {\textbf {y}}, {\textbf {x}}) =\pi (d \mid t, {\textbf {y}}, {\textbf {x}})\pi (t)$.

The marginal posterior density of the dose $\pi (d \mid {\textbf {y}}, {\textbf {x}})$ can be done by simulation accordingly with the following algorithm:

1. Generate a value $t^*$ of the prior distribution $\pi (t)$.
2. Generate a value of $(\alpha _{t^*},\beta _{t^*})$ from $\pi (\alpha _t, \beta _t \mid {\textbf {y}})$. It can be done using Gibbs sampler or using the Laplace approximation. In the last case, $(\alpha _{t^*},\beta _{t^*})$ is generated using the bivariate normal distribution (4).
3. Generate a value $\mu ^*$ of the normal distribution $\mathcal {N}(\bar{x}, \frac{s^2}{n})$. Use expression (5) with the inputs $\mu ^*$, $\alpha _{t^*}$ and $\beta _{t^*}$ for obtaining an estimated value of the dose $d^*$ and record it.
4. Repeat steps 1–4 many times (at least 10,000).

The simulated values $d^*$ from $\pi (d \mid {\textbf {y}}, {\textbf {x}})$ allow to obtain the median and credibility intervals of the dose received by the patient. It is worth to mention that when the Laplace approximation is used the density $\pi (d \mid {\textbf {y}}, {\textbf {x}})$ can also be obtained by numerical integration of $\pi (d,t\mid {\textbf {y}}, {\textbf {x}})$ with respect to *t*, see Supplementary material for details.

## Materials and results

Except for the three data sets chosen to test the models’ performance, we decided to use all of the data published by^11^ as calibration data. Radiation doses range from 0 to 3 Gy, with post-irradiation times ranging from 0.5 to 24 h. The Supplementary material contains all of the data used in this study as well as the R scripts used to obtain the results. Automatic microscopy was used to count the number of foci in 500 cells for each dose and time. The foci frequencies increase with increasing doses, as expected, but they decrease throughout time, reaching their lowest level after 24 h.

The calibration data were fitted to mixture Poisson models with $K=2,3,4$ and 5 components. Based on the Akaike information criterion (AIC^18^), we found that the best model was the mixture Poisson model with $K=4$ components. Moreover, we chose a model where the parameter $u_{k}$ is equal in all four components and from now on it will be denoted as *u*. It means that for further analysis the mixture Poisson model with 4 components was chosen with mean given by

$$\begin{aligned} \lambda _k(d,t)= c_k \cdot t^{u} + a_k \cdot t^{v_k} \cdot d,  k=1,2,3,4. \end{aligned}$$

leading to a 16 parameters model, the calibration coefficients, denoted as $(\hat{\omega },\hat{\theta })$. Supplementary Figure S1 shows the profile of the calibration surface. Following our strategy, the calibration procedure additionally estimates the 136 values of the variance-covariance matrix $\hat{\Sigma }_{\hat{\omega },\hat{\theta }}$. Therefore, the laboratory that performs the calibration (indicated in Fig. 3 as Laboratory (1) generates a total of 152 figures. Based on this data, a different (or same) laboratory (denoted in Fig. 3 as Laboratory (2) can check a patient’s blood sample and determine the radiation dose they received.

We used the test data from three data sets which weren’t included in the calibration data as an example of application to illustrate the methodology. Radiation doses were 0.75, 2, and 3 Gy, respectively, with post-irradiation durations of 4, 10, and 0.5 h. We assume that the laboratory that will conduct the patients’ blood tests will only provide aggregate foci data, such as the sample mean and variance, rather than raw data.

We chose to check the method’s robustness by using three different prior distributions ($\pi (t)$) for each test data set. The first prior is a uniform distribution on the intervals (0.25, 0.75), (3, 5), and (8, 12) that is centred at the known post-irradiation times of 0.5, 4, and 10 h, respectively. The second and third options are non-standard symmetric beta distributions (see Supplementary material for details) with parameters $\alpha =5,\beta =5$ and $\alpha =100,\beta =100$, on the same intervals as the uniform priors.

**Table 1:** Test data and dose estimates (mean and credible interval) obtained by Laplace approximation method.

Test data				Dose estimation			
Donor	Dose Gy	Time h	Foci mean ± SE	Prior distribution of time			
				Time interval h	Uniform	Beta (5, 5)	Beta (100, 100)
2	0.75	4.0	4.072 ± 0.230	(3, 5)	0.771 (0.567, 0.989)	0.773 (0.605, 0.948)	0.774 (0.622, 0.927)
1	2.00	10.0	4.036 ± 0.198	(8, 12)	1.38 (1.115, 1.662)	1.382 (1.159, 1.614)	1.383 (1.178, 1.591)
1	3.00	0.5	28.612 ± 0.525	(0.25, 0.75)	2.93 (2.044, 3.704)	2.955 (2.43, 3.434)	2.966 (2.785, 3.139)

**Figure 4:**
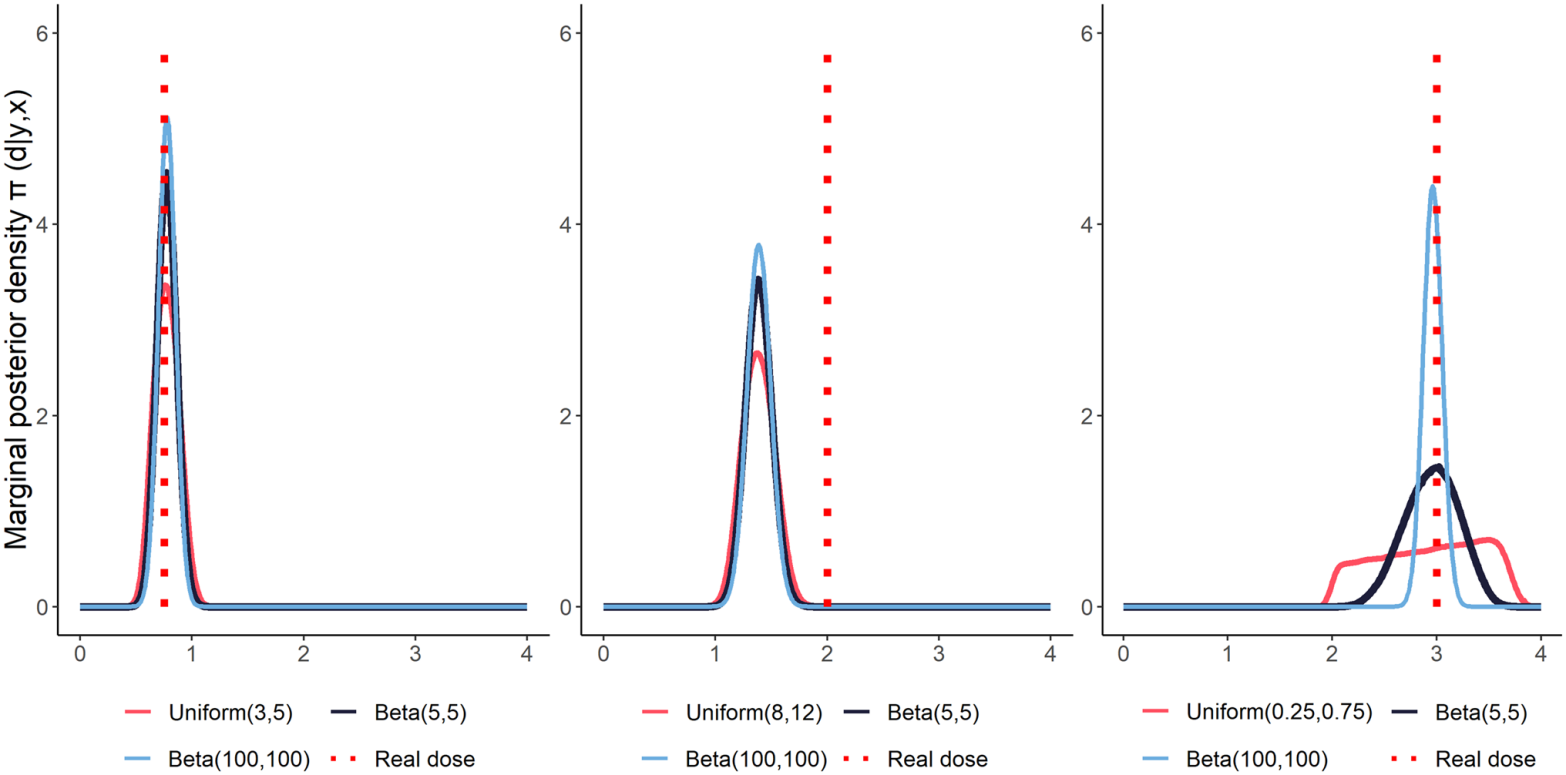
Marginal posterior densities $\pi (d \mid {\textbf {y}}, {\textbf {x}})$ for the test data obtained by Laplace approximation method. The prior distributions of the post-irradiation time are defined on the intervals: (3, 5), (8, 12), (0.25, 0.75) h.

To estimate the posterior marginal distribution for the dose $\pi (d \mid {\textbf {y}}, {\textbf {x}})$ we have applied the Laplace approximation and marginalising $\pi (d,t \mid {\textbf {y}}, {\textbf {x}})$. The analysis was performed using R and JAGS program and the codes can be found in Supplementary material. The results of the estimation are presented in Table 1 and the posterior densities $\pi (d \mid {\textbf {y}}, {\textbf {x}})$ can be seen in Fig. 4. The goodness of the Laplace approximation with respect to the Gibbs sampler has been checked providing the same results up to two decimal places (see Supplementary Table S1).

It can be seen that the model performs well for first and third test data, where the estimated dose is very close to the real one independently of the prior distribution of the time since irradiation considered. However, for second data set the dose is underestimated. Not surprisingly, the credible intervals are the narrowest for the prior beta distribution with $\alpha =100,\beta =100$.

## Discussion

The methods of biodosimetry are very useful in both small and large-scale accidents^19^. However, in both cases it is important to timely determine the radiation dose to those exposed, as it hopefully could lead to a prompt and effective treatment. Therefore the method of dose estimation should be quick and easy to use by people who provide help during emergencies^20^.

The main advantage of $\gamma$-H2AX assay, comparing to others biomarkers, is its speed, because it does not require a long process of culturing cells and can provide results within a few hours of receiving a blood sample^21^, what makes $\gamma$-H2AX biomarker a good tool for rapid triage in case of a mass casualty event^6^. Our model is mostly suggested for acute exposures. For chronic exposures, we can expect that the observed foci at a given post-irradiation time will be a mixture of the remaining induced DSB from multiple moments in the accident. Furthermore, we think that the applicability of $\gamma$-H2AX in determining chronic exposures appears to be very limited due to its transient nature.

In addition to the duration of the biological part of the procedure, we suggest also taking into account the speed of generating estimation results, because the more complex the model, the longer it takes to execute it. The Bayesian method proposed in this study, which is based on the Laplace approximation and numerical integration, is quick and accurate, and requires no simulations. When compared to the Gibbs sampling method, it produces nearly identical results, but the time required to estimate them is significantly longer. About 5000 iterations of the Gibbs sampling method were done, and the results took more than 39 h with these settings. Applying Laplace approximation allows for a significant reduction in the computation time needed to estimate the dose as the results of this method are immediate.

The present model is based on data previously published^11^ where PBMCs were exposed at doses ranging from 0 to 3 Gy and $\gamma$-H2AX foci were evaluated up to 24 h post-irradiation. However, the model allows to include doses that are not evaluated at all post-irradiation times. Therefore, it can include low doses up to 24 h that will not be analysed later, as well as high doses that will not be analysed in the first post-irradiation times, but later times such as 72 h. The ability to detect foci at late times after high-dose exposures for dose-assessment has been previously described^10,22,23^.

In addition, the model would need to be validated with real radiation exposures, either accidental cases or by evaluating the kinetics of foci disappearance after whole body irradiation in cancer patients. It has been described that in vivo data coming from cancer patients showed some differences to that obtained in vitro. For example, foci analysis on thyroid cancer patients treated with radioiodine indicated that while there was an agreement between the in vitro and in vivo calibration from 0 to 2 h post-treatment, at later times the foci observed in vivo were lower to those observed in vitro indicating a different progression in the DNA repair^24^.

In the present study we are proposing a model to be used under specific circumstances, accidents involving low-LET radiation types (X- or $\gamma$-rays). There is evidence that high-LET IR radiation produces more complex and clustered DNA damage^25^ than low-LET IR, and while low-LET radiation-induced DSBs are resolved quickly DSBs induced by high-LET is slower^26,27^. For this reason, the model here presented based on a Poisson mixture cannot be directly applied, and although Bayesian methods can be applied under different conditions, both the distribution of foci per cell, as well as their repair kinetics should be analysed previously to propose a dose estimation method based on the analysis of $\gamma$-H2AX for radiation exposures to high-LET.

The model proposed in this article could be extended further. One may consider for example a scenario of dose gradient exposure. Assuming that gradient exposures are heterogeneous irradiations in the radiation field, it seems that a mixture model could be appropriate to handle these data. However, the number of mixture components K would probably greatly increase. Nevertheless, it is quite likely that there may occurs some interference between the differential radiosensitivity of the leukocyte subsets and the dose gradient effect. It is a topic that requires more investigation through additional experiments, the findings of which can undoubtedly be interesting.

## Supplementary Information

Supplementary Information 1.

Supplementary Information 2.

## Data Availability

All data and computer code used for analysis during this study are included in Supplementary Information files.
