# Supplementary material for: Radiation dose estimation with time-since-exposure uncertainty using the γ-H2AX biomarker
Source: Sci Rep. 2022 Nov 18;12:19877. doi: 10.1038/s41598-022-24331-1 (PMC9674680; doi:10.1038/s41598-022-24331-1)
Supplement: Supplementary file 2 — Supplementary Information 2. [file 41598_2022_24331_MOESM2_ESM.pdf]

Supplemental material

Manuscript: Radiation dose estimation with time-since-exposure uncertainty using the γ-H2AX biomarker
Authors: Dorota Młynarczyk, Pedro Puig, Carmen Armero, Virgilio Gómez-Rubio, Joan F. Barquinero, Mònica Pujol-Canadell
Correspondence: dorotaanna.mlynarczyk@uab.cat

Perk's distribution

The Dirichlet distribution of order $K \geq 2$ is parameterised by a vector of positive real numbers $\alpha = (\alpha_1, \alpha_2, \ldots, \alpha_M)$ called concentration parameters. Its probability density function is given by

$$f(\omega_1, \ldots, \omega_K \mid \alpha) = \frac{\Gamma(\sum_{i=1}^{K} \alpha_i)}{\prod_{i=1}^{K} \Gamma(\alpha_i)} \prod_{i=1}^{K} \omega_i^{\alpha_i - 1},$$

where $\omega_i \geq 0$ and $\sum_{i=1}^{K} \omega_i = 1$. As a result of Alvares et al. (2018), we chose the Perks' prior. This is a Dirichlet distribution where all of the parameters are equal to $1/K$. This prior was proposed by Perks (1947), but Berger et al. (2015) obtained it as the reference distance prior.

Laplace approximation

Assume that $\boldsymbol{\phi}^* = (\hat{\omega}, \hat{\theta}) \in \mathbf{R}^p$ is the mode of the posterior density $\pi(\boldsymbol{\omega}, \boldsymbol{\theta} \mid \mathbf{y})$. Taking the second order Taylor expansion of $f(\boldsymbol{\phi}) = f(\boldsymbol{\omega}, \boldsymbol{\theta}) = \log(\pi(\boldsymbol{\omega}, \boldsymbol{\theta} \mid \mathbf{y}))$ centered on $\boldsymbol{\phi}^*$, we get:

$$f(\boldsymbol{\phi}) \approx f(\boldsymbol{\phi}^*) - \frac{1}{2}(\boldsymbol{\phi} - \boldsymbol{\phi}^*)^T \boldsymbol{H}^* (\boldsymbol{\phi} - \boldsymbol{\phi}^*),$$

where $\boldsymbol{H}^*$ is the Hessian matrix, the matrix of second-order partial derivatives of $f(\boldsymbol{\phi})$, evaluated at ϕ^*. Taking the exponential it gives,

$$\pi(\boldsymbol{\omega}, \boldsymbol{\theta} \mid \mathbf{y}) \propto \mathcal{L}(\mathbf{y} \mid \boldsymbol{\phi}^*) \pi(\boldsymbol{\phi}^*) \exp[-\frac{1}{2}(\boldsymbol{\phi} - \boldsymbol{\phi}^*)^T \boldsymbol{H}^* (\boldsymbol{\phi} - \boldsymbol{\phi}^*)].$$

The right side of the equation is a p-dimensional multivariate normal distribution, so the Laplace approximation provides a Gaussian approximation of the posterior, that is,

$$\pi(\boldsymbol{\omega}, \boldsymbol{\theta} \mid \mathbf{y}) \sim \mathcal{N}(\boldsymbol{\phi}^*, \boldsymbol{H}^{*-1}).$$

Thus it remains to determine the mode $\boldsymbol{\phi}^*$ of the posterior density $\pi(\boldsymbol{\omega}, \boldsymbol{\theta} \mid \mathbf{y})$. This procedure is known as maximum a posteriori (MAP) estimation and it is defined as,

$$\phi^* = \underset{(\omega, \theta)}{\arg\max}\, \mathcal{L}(\mathbf{y} \mid \boldsymbol{\omega}, \boldsymbol{\theta}) \pi(\boldsymbol{\omega}, \boldsymbol{\theta}).$$

Note that if flat distributions are chosen as prior distributions of model parameters, $\pi(\boldsymbol{\omega}, \boldsymbol{\theta}) \sim 1$, this estimator ϕ^* coincides with the maximum likelihood estimator (MLE), i.e. the model parameters which maximize the likelihood function.

A nonparametric Bayesian estimate of μ

Let **x** a vector of n independent observations coming from a distribution parametrized by its population mean μ, i.e. $f(x|\mu)$, with likelihood function,

$$\mathcal{L}(\mathbf{x}|\mu) = \prod_{i=1}^{n} f(x_i|\mu).$$

It is worth to mention that μ can also depend of other parameters. The posterior distribution of μ, given the prior $\pi(\mu)$, remains

$$\pi(\mu \mid \mathbf{x}) \propto \mathcal{L}(\mathbf{x} \mid \mu)\pi(\mu)\,.$$

It is known that for large n and under commonly satisfied regularity assumptions, the posterior density can be approximated as,

$$\pi(\mu \mid \mathbf{x}) \approx \mathcal{N}(\hat{\mu}, [I(\hat{\mu})]^{-1}), \tag{1}$$

where $\hat{\mu}$ is the maximum likelihood estimator (MLE) of μ and $I(\hat{\mu})$ is the Fisher information quantity evaluated at $\hat{\mu}$. It is remarcable that this asymptotic result is independent of the chosen prior $\pi(\mu)$ (see Berger et al. (2015), Result 8, p. 224).

It is said that distributions satisfy the Gauss' principle if the MLE of μ is the sample mean, i.e. $\hat{\mu} = \bar{x}$ (see Puig (2008) and the references therein). For instance, the Gauss' principle is satisfied by the Normal, Poisson, and Negative Binomial distributions. In particular, it can be demonstrated that the finite mixtures of Poisson distributions satisfy the Gauss' principle using the findings of Bondesson (1997). For these distributions expression (1) remains,

$$\pi(\mu \mid \mathbf{x}) \approx \mathcal{N}(\bar{x}, \hat{\sigma}^2/n),$$

where $\hat{\sigma}^2$ is the MLE of the variance $Var(x_i) = \sigma^2$. Finally, we replace $\hat{\sigma}^2$ by the sample variance that is a robust estimator of σ^2, obtaining,

$$\pi(\mu \mid \mathbf{x}) \approx \mathcal{N}(\bar{x}, s^2/n).$$

Due to technical limitations or a lack of a sufficient sample size, Laboratory 2 could be unable to fit a K-mixture of Poisson distributions. However, our Bayesian approach for calculating the received dose of radiation can be used by simply recording the mean number of foci found in test data, $\bar{x}$, and its standard deviation s (or variance s^2).

Ratio of two dependent normal variables

The following result can be found in Pham-Gia et al. (2006):

Theorem 1 *Let* $(X, Y) \sim \mathcal{N}_2(\mu_X, \mu_Y; \sigma_X, \sigma_Y; \rho)$. *Then the density of* $W = X/Y$ *is*

$$f_W(w; \mu_X, \mu_Y; \sigma_X, \sigma_Y; \rho) = K_2 \frac{2(1-\rho^2)\sigma_X^2\sigma_Y^2}{\sigma_Y^2 w^2 - 2\rho\sigma_X\sigma_Y w + \sigma_X^2}\, {}_1F_1(1; 1/2; \theta_2(w)),$$

$-\infty < w < \infty$,*where*
$\theta_2(w) = \frac{[-\sigma_Y^2\mu_X w + \rho\sigma_X\sigma_Y(\mu_Y w + \mu_X) - \mu_Y\sigma_X^2]^2}{2\sigma_X^2\sigma_Y^2(1-\rho^2)(\sigma_Y^2 w^2 - 2\rho\sigma_X\sigma_Y w + \sigma_X^2)} \geq 0,$
$K_2 = \frac{1}{2\pi\sigma_X\sigma_Y\sqrt{1-\rho^2}} \exp(-\frac{\sigma_Y^2\mu_X^2 - 2\rho\sigma_X\sigma_Y\mu_X\mu_Y + \mu_Y^2\sigma_X^2}{2(1-\rho^2)\sigma_X^2\sigma_Y^2})$ *and*
${}_1F_1(\alpha, \gamma; z) = \sum_{k=0}^{\infty} \frac{(\alpha,k)}{(\gamma,k)} \frac{z^k}{k!}$ *where* $(\alpha, k) = \alpha(\alpha+1)\ldots(\alpha+k-1) = \Gamma(\alpha+k)/\Gamma(\alpha)$ *with* $(\alpha, 0) = 1$.

In our case the dose is calculated as,

$$d = \frac{\mu - \alpha_t}{\beta_t} = \frac{X}{Y},$$

where $\alpha_t = \sum_{k=1}^{K} \omega_k \cdot c_k \cdot t^{u_k}$, $\beta_t = \sum_{k=1}^{K} \omega_k \cdot a_k \cdot t^{v_k}$. Moreover, μ follows a normal distribution $\mathcal{N}(\bar{\mathbf{x}}, \frac{s^2}{n})$, where $\bar{\mathbf{x}}$ is the mean number of foci found in test data, s is the sample standard error and n is the number of observations in test data. Note that X and Y satisfy the assumptions of the theorem where, $\mu_X = \mu - \alpha_t$, $\mu_Y = \beta_t$, $\sigma_X = \sqrt{\frac{s^2}{n} + \mathrm{Var}(\alpha_t)}$, $\sigma_Y = \sqrt{\mathrm{Var}(\beta_t)}$, $\rho = \frac{\mathrm{Cov}(X,Y)}{\sigma_X \sigma_Y}$ and $\mathrm{Cov}(X,Y) = \mathrm{Cov}(\mu - \alpha_t, \beta_t) = \mathrm{Cov}(\mu, \beta_t) - \mathrm{Cov}(\alpha_t, \beta_t) = -\mathrm{Cov}(\alpha_t, \beta_t)$.

The function ${}_1F_1(\alpha, \gamma; z)$ is the Kummer's classical confluent hypergeometric function of first kind. For $\alpha = 1$ and $\gamma = 1/2$ it can be expressed as,

$${}_1F_1(1, 1/2; \theta_2(w)) = \exp(\theta_2(w))\sqrt{\pi}\sqrt{\theta_2(w)}\,\mathrm{erf}(\sqrt{\theta_2(w)}) + 1.$$

Non-standard beta distribution

The non-standard beta distribution is a parametrized by two positive shape parameters $\alpha, \beta > 0$. Its probability density function is given by

$$f(x) = \frac{(x-p)^{\alpha-1}(q-x)^{\beta-1}}{B(\alpha,\beta)(q-p)^{\alpha+\beta-1}}, \qquad p \le x \le q,$$

where p and q are the lower and upper bounds, respectively, of the distribution and $B(\alpha, \beta)$ is the beta function defined by the integral

$$B(\alpha, \beta) = \int_0^1 t^{\alpha-1}(1-t)^{\beta-1}\mathrm{d}t.$$

The expectation of the non-standard beta distribution is $p + \frac{\alpha}{\alpha+\beta}(q-p)$ and the variance is $\frac{\alpha\beta}{(\alpha+\beta)^2(\alpha+\beta+1)}(q-p)^2$.

Supplemental References

Alvares, D., Armero, C., and Forte, A. (2018). What Does Objective Mean in a Dirichlet-multinomial Process? *International Statistical Review*, **86**:4, 106–118.

Bondesson, L. (1997). A generalization of Poincare's characterization of exponential families. *J. Statist. Plann. Inference*, **63**:2, 147–155.

Perks, W. (1947). Some observations on inverse probability including a new indifference rule. *J. Inst. Actuaries*, **73**(2), 285–334.

Puig, P. (2008). A note on the harmonic law: A two-parameter family of distributions for ratios. *Statistics & Probability Letters*, **78**(3), 320–326.

Test data				Dose estimation			
Donor	Dose Gy	Time h	Foci Mean ± SE	Prior distribution of time			
				Time interval h	Uniform	Beta(5,5)	Beta(100,100)
Laplace approximation							
1	3.00	0.5	28.612 ± 0.525	(0.25,0.75)	2.93 (2.044,3.704)	2.955 (2.43,3.434)	2.966 (2.785,3.139)
2	0.75	4.0	4.072 ± 0.230	(3,5)	0.771 (0.567,0.989)	0.773 (0.605,0.948)	0.774 (0.622,0.927)
1	2.00	10.0	4.036 ± 0.198	(8,12)	1.38 (1.115,1.662)	1.382 (1.159,1.614)	1.383 (1.178,1.591)
Gibbs sampler							
1	3.00	0.5	28.612 ± 0.525	(0.25,0.75)	2.933 (2.05,3.705)	2.959 (2.432,3.432)	2.968 (2.801,3.137)
2	0.75	4.0	4.072 ± 0.230	(3,5)	0.774 (0.574,0.984)	0.773 (0.608,0.95)	0.773 (0.621,0.919)
1	2.00	10.0	4.036 ± 0.198	(8,12)	1.381 (1.124,1.656)	1.379 (1.158,1.602)	1.384 (1.191,1.584)

Table S1: Test data and dose estimates (mean and credible interval) obtained by Laplace approximation method and Gibbs sampler.

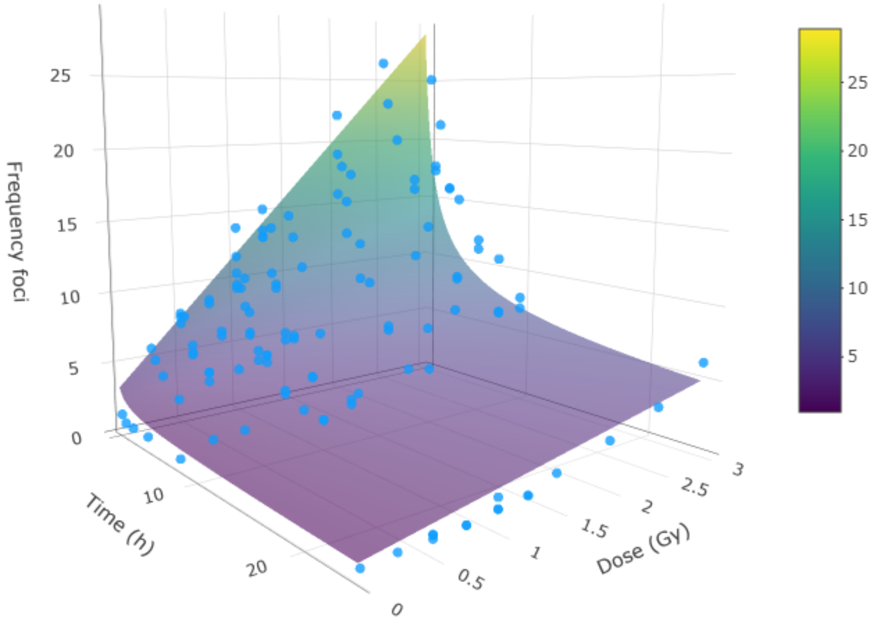

Figure S1: Surface calibration model, calibration data points marked in blue.

R code for the article ***Radiation dose estimation with time-since-exposure uncertainty using the γ-H2AX biomarker***

Program for preparing data and defining functions

```
#Calibration data

library("readxl")

data <- read_excel("./data.xlsx")

#Rows 8,63,105 were chosen as test data

calibration_data<-data[-c(8,63,105),]

test_data<-data[c(8,63,105),]


#Preparing data for analysis

dose<-calibration_data$dose

time<-calibration_data$time

d<-dim(calibration_data)[1]

nfoci_max<-as.integer(dim(calibration_data)[2]-5)

ncel<-rowSums(calibration_data[-c(1,2,3,4)])

#Saving calibration data as vectors

calib_data<-calib_dose<-calib_time<-numeric()

for(j in 1:d){

  for (i in 0:nfoci_max){

    calib_data<-c(calib_data,rep(i,calibration_data[j,i+5]))

    calib_dose<-c(calib_dose,rep(dose[j],calibration_data[j,i+5]))

    calib_time<-c(calib_time,rep(time[j],calibration_data[j,i+5]))

  }}

#Calculating mean and se of test data

#test_data[,c("mean","se")]<-NA

test_freq<-numeric()

mean_all<-se_all<-NULL;

for(j in 1:dim(test_data)[1]){

  for (i in 0:nfoci_max){

    test_freq<-c(test_freq,rep(i,test_data[j,i+5]))

  }
```

```
mean_all<-c(mean_all,mean(test_freq))

se_all<-c(se_all,sqrt(var(test_freq)/length(test_freq)))

test_freq<-NULL

}

test_data$mean<-mean_all

test_data$se<-se_all

#save(calib_data,calib_dose, calib_time, dose, time, calibration_data,test_data, file="data_all.Rdata")


#Functions

#Defining alpha_t, beta_t (Article section 2.1)

alpha<-function(t){(omega1*c1+omega2*c2+omega3*c3+(1-omega1-omega2-omega3)*c4)*t^u}

beta<-function(t){omega1*a1*t^v1+omega2*a2*t^v2+omega3*a3*t^v3+(1-omega1-omega2-omega3)*
a4*t^v4}


#Calculating derivatives (for gradient: expression(1) in the article)

derivativesf1<-deriv(~((omega1*c1+omega2*c2+omega3*c3+(1-omega1-omega2-omega3)*c4)*t^u),


c("omega1","omega2","omega3","c1","c2","c3","c4","a1","a2","a3","a4","u","v1","v2","v3","v4"),

                 function(t,omega1,omega2,omega3,c1,c2,c3,c4,a1,a2,a3,a4,u,v1,v2,v3,v4){})

derivativesf2<-deriv(~(omega1*a1*t^v1+omega2*a2*t^v2+omega3*a3*t^v3+(1-omega1-omega2-omeg
a3)*a4*t^v4),


c("omega1","omega2","omega3","c1","c2","c3","c4","a1","a2","a3","a4","u","v1","v2","v3","v4"),

                 function(t,omega1,omega2,omega3,c1,c2,c3,c4,a1,a2,a3,a4,u,v1,v2,v3,v4){})

#Defining error function (needed for Kummer’s classical confluent hypergeometric function)

erf <- function(x) 2*pnorm(x*sqrt(2))-1


#Calculating density of the ratio of two dependent normal variables (see supplemental material)

density_dt<-function(w,mu_X,mu_Y,sigma_X,sigma_Y,rho){

  theta<-(-sigma_Y^2*mu_X*w+rho*sigma_X*sigma_Y*(mu_Y*w+mu_X)-mu_Y*sigma_X^2)^2/

    (2*sigma_X^2*sigma_Y^2*(1-rho^2)*(sigma_Y^2*w^2-2*rho*sigma_X*sigma_Y*w+sigma_X^2))

  C<-1/(2*pi*sigma_X*sigma_Y*sqrt(1-rho^2))*
exp(theta-(sigma_Y^2*mu_X^2-2*rho*sigma_X*sigma_Y*mu_X*mu_Y+mu_Y^2*sigma_X^2)/(2*(1-rho^
2)*sigma_X^2*sigma_Y^2))*sqrt(pi)*sqrt(theta)*erf(sqrt(theta))+1/(2*pi*sigma_X*sigma_Y*sqrt(1-rho^
2))*exp(-(sigma_Y^2*mu_X^2-2*rho*sigma_X*sigma_Y*mu_X*mu_Y+mu_Y^2*sigma_X^2)/(2*(1-rho^2
)*sigma_X^2*sigma_Y^2))
```

```
  den<-C*(2*(1-rho^2)*sigma_X^2*sigma_Y^2)/(sigma_Y^2*w^2-2*rho*sigma_X*sigma_Y*w
+sigma_X^2)
  return(den)
}


#Calculating posterior distribution of dose conditioned to time: π(d|t,y,x) (Article section 2.2)
posterior<-function(t,w,mu_donor,se_donor){
  alpha_t<-alpha(t)
  beta_t<-beta(t)
  gradf1 <- attr(derivativesf1(t,omega1=omega1,omega2=omega2 ,
omega3=omega3,c1=c1,c2=c2,c3=c3,c4=c4,a1=a1,a2=a2,a3=a3,a4=a4,
                         u=u,v1=v1,v2=v2,v3=v3,v4=v4),"gradient")
  gradf2 <- attr(derivativesf2(t,omega1=omega1,omega2=omega2 ,
omega3=omega3,c1=c1,c2=c2,c3=c3,c4=c4,a1=a1,a2=a2,a3=a3,a4=a4,
                         u=u,v1=v1,v2=v2,v3=v3,v4=v4),"gradient")
  grad<-as.matrix(rbind(gradf1,gradf2))
  sigma_t=grad%*%varcov%*%t(grad)
  alpha_var<-sigma_t[1,1]
  beta_var<-sigma_t[2,2]
  cov<-sigma_t[2,1]


  mu_x<-mu_donor-alpha(t)
  mu_y<-beta(t)
  sigma_x<-sqrt(se_donor^2+alpha_var)
  sigma_y<-sqrt(beta_var)
  rho<-(-cov)/(sigma_x*sigma_y)
  return(density_dt(w,mu_x,mu_y,sigma_x,sigma_y,rho))
}
#save(alpha,beta,derivativesf1,derivativesf2,posterior,density_dt,erf, file="functions.Rdata")
```

Program for calibration

Laplace approximation

```
#Program for calibration part of the model using Laplace approximation method

load("data_all.Rdata")

##Loglikelihood function for model with 4 components

data_out<-as.matrix(calibration_data[-c(1,2,3,4)])

d<-dim(data_out)[1]

n<-dim(data_out)[2]

par=numeric(16)

set.seed(6453)

loglike<-function(par){

 loglike<-0

 for(j in 1:d){

  lam1<-par[4]*time[j]^par[12]+par[8]*dose[j]*time[j]^par[13]

  lam2<-par[5]*time[j]^par[12]+par[9]*dose[j]*time[j]^par[14]

  lam3<-par[6]*time[j]^par[12]+par[10]*dose[j]*time[j]^par[15]

  lam4<-par[7]*time[j]^par[12]+par[11]*dose[j]*time[j]^par[16]

  for(i in 1:n){

   loglike<-loglike+

    log(par[1]*dpois(i-1,lam1)

      +par[2]*dpois(i-1,lam2)

      +par[3]*dpois(i-1,lam3)

      +(1-par[1]-par[2]-par[3])*dpois(i-1,lam4))*data_out[j,i]

  }

 }

 -loglike}


#Initial values

inito<-c(0.22,0.28,0.35)

initc<-c(0.01,0.24,2.48,11.88)

inita<-c(0.63,4.36,9.12,9.91)

initu<--0.31

initv<-c(-0.79,-0.71,-0.51,-0.29)
```

```
#Finding Laplace approximation of the posterior π(ω,θ|y)-multivariate normal distribution

MLE<-nlm(loglike,p=c(inito,initc,inita, initu, initv),hessian=TRUE, iterlim = 150, steptol = 1e-50, gradtol = 1e-100)

MLE

#save(MLE, file="MLE.Rdata")

#round(MLE$minimum,2)

#round(MLE$estimate,2)
```

Gibbs sampler

```
#Program for calibration part of the model using MCMC method

library(rjags)

load("./data_all.Rdata")

set.seed(6453)

n.grp=4

# Initial grouping using K-means

centers <- sort(kmeans(calib_data, centers = n.grp)$centers)

groupid <- kmeans(calib_data, centers = centers)$cluster


#Data

dataF4.jags <- list(x= calib_data, M=length(calib_data),

          cal_dose=calib_dose, cal_time=calib_time,

          K = n.grp, alpha=rep(0.25,n.grp))

#Initial values

inits4.jags<-list(omega=c(0.29, 0.3,0.27, 0.14), c=c(0.06,0.86,2.58,9.73), a=c(0.73,4.39,10.6,12.63),u=-0.42,v=c(-0.81,-0.84,-0.48,-0.23), group = groupid)


#Model definition in BUGS (TO BE SAVED as ´mix_poisson.bug´)

model {

# Observations

#Each obs belongs to one component of mixture

 for(i in 1:M) {

  group[i] ~ dcat(omega[])

 }
```

```
#Calibration data

# Observations follow Poisson distribution with parameter lambda[i]=c·t_i^u+a·t_i^v·d_i

  for(i in 1:M) {

    x[i] ~ dpois(lambda[i])

    lambda[i]<-c[group[i]]*cal_time[i]^u+a[group[i]]*cal_time[i]^v[group[i]]*cal_dose[i]

  }

#Parameters flat distributions

  for (k in 1:K) {

    c[k] ~ dunif(-10000,10000)

    a[k] ~ dunif(-10000,10000)

    v[k] ~ dunif(-10000,10000)

  }

  u ~ dunif(-10000,10000)


#Weights ´omega´: dirichlet(alpha-defined in jags.data)

  omega[1:K] ~ ddirch(alpha[])

}

#Model JAGS

model4.jags <- jags.model("./mix_poisson.bug", data4.jags, inits4.jags,  n.chains = 2)

update(model4.jags, 500)

results4.jags <- coda.samples(model4.jags, c("omega", "c","a","u","v"),

                              n.iter = 5000, thin = 2)

#Results

summary(results4.jags)

#save(resultsF4.jags, inits4.jags, modelF4.jags, centers, file="./results_calib_MCMC.Rdata")


#Traceplots

library(MCMCvis)

MCMCtrace(resultsF4.jags,ISB = FALSE, exact = TRUE,pdf = TRUE,filename = 'traces')
```

Program for dose estimation

Laplace approximation

```
#Program for dose estimation using Laplace approximation method in the calibration part

load("functions.Rdata")

load("MLE.Rdata")

set.seed(6453)

library(extraDistr)

#Renaming results from calibration part

omega1<-MLE$estimate[1];omega2<-MLE$estimate[2];omega3<-MLE$estimate[3];

c1<-MLE$estimate[4];c2<-MLE$estimate[5];c3<-MLE$estimate[6];c4<-MLE$estimate[7];

a1<-MLE$estimate[8];a2<-MLE$estimate[9];a3<-MLE$estimate[10];a4<-MLE$estimate[11];

u<-MLE$estimate[12]

v1<-MLE$estimate[13];v2<-MLE$estimate[14];v3<-MLE$estimate[15];v4<-MLE$estimate[16];

varcov<-solve(MLE$hessian)


#PATIENT DATA:

patient_mean=28.612

patient_se=0.525

#TIME INTERVAL to be defined:

lower_time<-0.25

upper_time<-0.75


#Prior time distribution: UNIFORM

uniform<-Vectorize(function(w) sapply(w, function(w) (integrate(function(t)
(posterior(t,w,mu_donor=patient_mean,se_donor=patient_se)/(upper_time-lower_time)),

                                        lower=lower_time,upper=upper_time)$value)))

dose_unif<-integrate(function(z) z*uniform(z),-Inf,Inf, subdivisions=350)$value

distr_unif<-function(t){integrate(uniform,-Inf,t,subdivisions = 350)$value}

CIL_unif<-uniroot(function(x){distr_unif(x)-0.025},c(-2,dose_unif))$root

CIU_unif<-uniroot(function(x){distr_unif(x)-0.975},c(dose_unif,5))$root
```

```
#Prior time distribution: BETA(5,5) (Non-standard beta distribution: see supplemental material for details)

beta55<-Vectorize(function(w) sapply(w, function(w)

  (integrate(function(t) (posterior(t,w,mu_donor=patient_mean,se_donor=patient_se)*dnsbeta(t, 5, 5, min = lower_time, max = upper_time, log = FALSE)), lower=lower_time,upper=upper_time)$value)))

dose_beta55<-integrate(function(z) z*beta55(z),-Inf,Inf,subdivisions = 350)$value

distr_beta55<-function(t){integrate(beta55,-Inf,t,subdivisions = 350)$value}

CIL_beta55<-uniroot(function(x){distr_beta55(x)-0.025},c(-2,dose_beta55))$root

CIU_beta55<-uniroot(function(x){distr_beta55(x)-0.975},c(dose_beta55,5))$root


#Prior time distribution: BETA(100,100) (Non-standard beta distribution: see supplemental material for details)

beta100<-Vectorize(function(w) sapply(w, function(w)

   (integrate(function(t) (posterior(t,w,mu_donor=patient_mean,se_donor=patient_se)*dnsbeta(t, 100, 100, min = lower_time, max = upper_time, log = FALSE)), lower=lower_time,upper=upper_time)$value)))

dose_beta100<-integrate(function(z) z*beta100(z),-Inf,Inf,subdivisions = 350)$value

distr_beta100<-function(t){integrate(beta100,-Inf,t,subdivisions = 350)$value}

CIL_beta100<-uniroot(function(x){distr_beta100(x)-0.025},c(-2,dose_beta100))$root

CIU_beta100<-uniroot(function(x){distr_beta100(x)-0.975},c(dose_beta100,5))$root


#Preparing table with results

results<-data.frame(rbind(round(c(NA,patient_mean,patient_se),3),

                 c("Dose_est", "CIL","CIU"),

                 round(c(dose_unif,CIL_unif,CIU_unif),3),

                 round(c(dose_beta55,CIL_beta55,CIU_beta55),3),

                 round(c(dose_beta100,CIL_beta100,CIU_beta100),3)))

results<-cbind(c("Data","","Uniform","Beta(5,5)","Beta(100,100)"),results)

colnames(results) <- c("","",'Mean', 'SE')

results
```

Gibbs sampler

```
#Program for dose estimation using MCMC method in the calibration part

set.seed(6453)

load("./results_calib_MCMC.Rdata")
```

```
#Defining alpha_t, beta_t (Article section 2.1)

alpha_f<-function(t,c1,c2,c3,c4,omega1,omega2,omega3,omega4,u){(omega1*c1+omega2*c2+omega3*c3+omega4*c4)*t^u}

beta_f<-function(t,a1,a2,a3,a4,omega1,omega2,omega3,omega4,v1,v2,v3,v4){omega1*a1*t^v1+omega2*a2*t^v2+omega3*a3*t^v3+omega4*a4*t^v4}

library(rjags)

mcmc_samples<-mcmc(do.call(rbind, results4.jags))

n_sim=dim(mcmc_samples)[1]


#PATIENT DATA:

patient_mean=28.612

patient_se=0.525

#TIME INTERVAL to be defined:

lower_time<-0.25

upper_time<-0.75


#Prior time distribution: UNIFORM

r_x<-rnorm(n_sim, mean=patient_mean, sd=as.numeric(patient_se))

time=runif(n_sim,min=lower_time,max=upper_time)

dose<-NULL;time<-NULL

for (j in 1:n_sim) {

  sim_data<-as.numeric(mcmc_samples[j,])


alpha<-alpha_f(t=time[j],c1=sim_data[5],c2=sim_data[6],c3=sim_data[7],c4=sim_data[8],omega1=sim_data[9],omega2=sim_data[10],omega3=sim_data[11],omega4=sim_data[12],u=sim_data[13])

  beta<-beta_f(t=time[j],
a1=sim_data[1],a2=sim_data[2],a3=sim_data[3],a4=sim_data[4],omega1=sim_data[9],omega2=sim_data[10],omega3=sim_data[11],omega4=sim_data[12],v1=sim_data[14],v2=sim_data[15],v3=sim_data[16],v4=sim_data[17])

  dose[j]<-(r_x[j]-alpha)/beta

}

dose_unif=mean(dose)

CIL_unif=quantile(dose,0.025)

CIU_unif=quantile(dose,0.975)
```

```
#Prior time distribution: BETA(5,5) (Non-standard beta distribution: see supplemental material for details)

library(extraDistr)

dose<-NULL;time<-NULL

r_x<-rnorm(n_sim, mean=patient_mean, sd=as.numeric(patient_se))

time=rnsbeta(n_sim, 5, 5, min = lower_time, max = upper_time)

for (j in 1:n_sim) {

  sim_data<-as.numeric(mcmc_samples[j,])


alpha<-alpha_f(t=time[j],c1=sim_data[5],c2=sim_data[6],c3=sim_data[7],c4=sim_data[8],omega1=sim_data[9],omega2=sim_data[10],omega3=sim_data[11],omega4=sim_data[12],u=sim_data[13])

  beta<-beta_f(t=time[j],
a1=sim_data[1],a2=sim_data[2],a3=sim_data[3],a4=sim_data[4],omega1=sim_data[9],omega2=sim_data[10],omega3=sim_data[11],omega4=sim_data[12],v1=sim_data[14],v2=sim_data[15],v3=sim_data[16],v4=sim_data[17])

  dose[j]<-(r_x[j]-alpha)/beta

}

dose_beta55=mean(dose)

CIL_beta55=quantile(dose,0.025)

CIU_beta55=quantile(dose,0.975)


#Prior time distribution: BETA(100,100) (Non-standard beta distribution: see supplemental material for details)

dose<-NULL;time<-NULL

r_x<-rnorm(n_sim, mean=patient_mean, sd=as.numeric(patient_se))

time=rnsbeta(n_sim, 100, 100, min = lower_time, max = upper_time)

for (j in 1:n_sim) {

  sim_data<-as.numeric(mcmc_samples[j,])


alpha<-alpha_f(t=time[j],c1=sim_data[5],c2=sim_data[6],c3=sim_data[7],c4=sim_data[8],omega1=sim_data[9],omega2=sim_data[10],omega3=sim_data[11],omega4=sim_data[12],u=sim_data[13])

  beta<-beta_f(t=time[j],
a1=sim_data[1],a2=sim_data[2],a3=sim_data[3],a4=sim_data[4],omega1=sim_data[9],omega2=sim_data[10],omega3=sim_data[11],omega4=sim_data[12],v1=sim_data[14],v2=sim_data[15],v3=sim_data[16],v4=sim_data[17])

  dose[j]<-(r_x[j]-alpha)/beta

}
```

```
dose_beta100=mean(dose)

CIL_beta100=quantile(dose,0.025)

CIU_beta100=quantile(dose,0.975)


#Preparing table with results

results<-data.frame(rbind(round(c(NA,patient_mean,patient_se),3),

                c("Dose_est", "CIL","CIU"),

                round(c(dose_unif,CIL_unif,CIU_unif),3),

                round(c(dose_beta55,CIL_beta55,CIU_beta55),3),

                round(c(dose_beta100,CIL_beta100,CIU_beta100),3)))

results<-cbind(c("Data","","Uniform","Beta(5,5)","Beta(100,100)"),results)

colnames(results) <- c("","",'Mean', 'SE')

results
```
